# Enhanced small intestinal organoid-derived epithelial cell adhesion and growth in organ-on-a-chip devices†

**DOI:** 10.1039/d4ra08290g

**Published:** 2025-02-05

**Authors:** Federica Quacquarelli, Sergio Davila, Jasin Taelman, Jordi Guiu, Maria Antfolk

**Affiliations:** a Department of Biomedical Engineering, Lund University Lund Sweden maria.antfolk@bme.lth.se; b Cell Plasticity and Regeneration Group, Regenerative Medicine Program, Institut d'Investigació Biomèdica de Bellvitge-IDIBELL L'Hospitalet de Llobregat Spain; c Program for Advancing the Clinical Translation of Regenerative Medicine of Catalonia, P-CMR[C] L'Hospitalet de Llobregat Spain

## Abstract

Organ-on-a-chip devices are predominately made of the polymer polymethylsiloxane (PDMS), exhibiting several attractive properties *e.g.*, transparency, gas permeability, and biocompatibility. However, the attachment of cells to this polymer has proven challenging, especially for delicate primary cells *e.g.*, small intestinal organoid-derived epithelial cells. Hence, a need to functionalize and coat the surface has arisen to render it more hydrophilic and improve its ability to support cell adhesion and growth. While previous research has demonstrated some successful results in culturing primary cells, no comprehensive and comparative protocol has been proposed. Here, we provide a protocol for enhanced small intestinal organoid-derived epithelial cell adhesion and growth on PDMS and plastics, assessing both PDMS surface functionalization, adhesion protein coating as well as medium selection. We assess PDMS functionalization using (3-aminopropyl)trimethoxysilane (APTMS) or polyethyleneimine-glutaraldehyde (PEIGA), and adhesion protein coating using various Laminins, Collagen I, Matrigel, or mixtures thereof. Finally, we assess the use of two different medium compositions including growth factors EGF, Noggin and R-spondin1 (ENR medium) alone or combined with the two small molecules CHIR99021 and valproic acid (CV medium). We envision that our results will be useful for further attempts in emulating the small intestine using plastic- or PDMS-based devices for organs-on-a-chip development.

## Abstract 2

PDMS functionalization and adhesion protein coatings in combination with different medium formulations was assessed for the adhesion and growth of intestinal epithelial cells.

## Graphical abstract

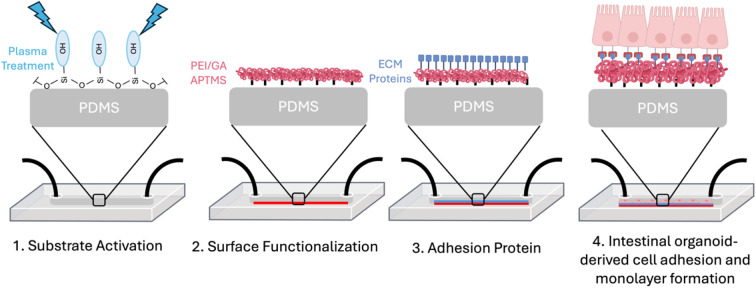

## Introduction

Intestinal epithelial cells can be cultured long-term as organoids in three-dimensional scaffolds.^1,2^ These self-organized units contain all the major cell types of the intestinal epithelium, and comprise near-physiological systems that have proven immensely useful for addressing questions on intestinal biology in homeostasis and disease.^3^ Intestinal organoids are especially attractive for genetic studies and have been used extensively to study the effect of individual genes *in vitro*.^4–6^ Even though these organoids have proven very useful and versatile they inherently possess limitations, as they comprise closed structures. The apical side of the intestinal epithelium is facing the hollow closed-of lumen of the organoid, while the basolateral side faces outwards. This complicates interaction or translocation studies where access to the apical side is important.^7^ To facilitate such studies protocols for culturing apical-out organoids have been developed.^8^ Even though this has provided means to ease nutrient uptake, or infection studies, these organoids still represent a static system not fully emulating the dynamic processes of the *in vivo* intestine.

The organs-on-a-chip technology has emerged as an attractive complementary approach to organoids and the usefulness of this technique has been elegantly demonstrated.^9^ A few intestine-on-a-chip devices have been proposed and have demonstrated the importance of dynamic culture conditions including a fluid flow, simulating both the movement of food matter as well as the underlaying blood flow, and the ability to simulate the mechanical motion of peristalsis.^10–12^ Most previous intestine-on-a-chip systems have been based on cell lines, of which the colorectal adenocarcinoma cell line Caco-2 is most commonly used. Even though these systems have provided some interesting knowledge on the intestinal biology, they can never fully recapitulate the intricated biological function of the healthy intestine. Cancer cell lines inherently represent a diseased cell state of immortalized cells, and often fail to reproduce the natural heterogeneity of the healthy intestine, including cell fate decisions and stem cell maintenance. Cancer cells also harbor mutations that change their phenotype compared to healthy cells. An example of this is the Caco-2 cell line, which has mutations in the APC protein that normally regulates canonical WNT signaling, which is, important for intestinal stem cell self-renewal. The colon origin of this cell line also makes it less suitable for emulating the small intestine. A few small intestine-on-a-chip devices utilizing healthy cells have been proposed recently, demonstrating the possibility to use both iPSCs and primary organoid-derived intestinal epithelial cells in these dynamic microphysiological systems.^13–17^

Most of these systems are based on a similar design where the intestinal epithelial cells are culture on a porous membrane, fabricated in the polymer polydimethylsiloxane (PDMS), that separates an upper and a lower channel. The upper channel harboring the intestinal epithelial cells represents the intestinal lumen and the lower channel on the opposite side of the porous membrane represents a blood vessel.^18^ The flexibility of PDMS is important especially for simulating the mechanical peristaltic motion of the intestine.^10^ However, PDMS is inherently a hydrophobic polymer that makes cell adhesion more challenging. While many cell lines easily adhere to bare PDMS or PDMS that has simply been coated with a given adhesion protein, some primary cells require prior functionalization to realize more strongly bound adhesion proteins in order to be able to adhere and fully cover a PDMS surface.^19^ Given that small intestinal epithelial cells cannot readily be cultured on uncoated surfaces these delicate cells require specific functionalization and adhesion opportunities to be able to adhere and spread in both ordinary cell culture plastic vessels and on PDMS surfaces. Previous small intestine-on-a-chip devices have focused on functionalization using (3-aminopropyl)trimethoxysilane (APTMS) or sulfosuccinimidyl 6 (4′-azido-2′-nitrophenyl-amino) hexanoate (sulfo-SANPAH), and subsequently coated the membrane with a collagen I and Matrigel mixture without showing that this would be the optimal solution.^13,15,16,20^ Functionalization using APTMS silanizes the PDMS and generates functional amine groups on the surface, and sulfo-SANPAH functions as a crosslinker that contains amine-reactive groups that enhances the adsorption of cell adhesion proteins.^21^

In this paper we provide an protocol for generating fully covering small intestinal organoid-derived monolayers on both PDMS and cell culture plastic surfaces. We have explored different ways of functionalizing the PDMS surfaces, different adhesion proteins as well as different medium formulations to understand which combinations that can support cell adhesion and the formation of 100% confluent monolayers. We envision that this paper will be useful both for scientists wishing to culture small intestinal organoid-derived monolayers in microfluidic devices and researchers that wish to culture these cells in static monolayer cultures on cell culture plastics.

## Results and discussion

**Fig. 1:**
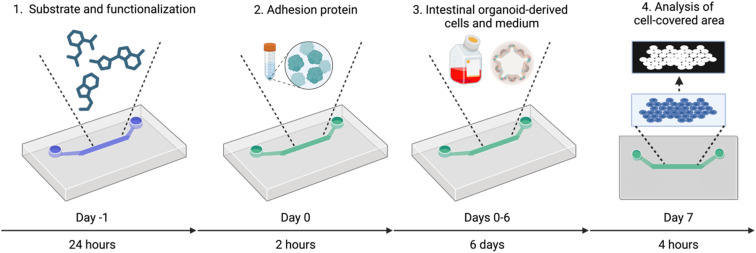
Step by step illustration of the experimental and analysis procedure involved in establishing the intestinal organoid-derived chip-based monolayer cultures. Created with http://biorender.com/.

In this paper we provide a protocol for generating small intestinal organoid-derived monolayer culture on different substrate surfaces for organ-on-a-chip applications (Fig. 1). Firstly, we fabricated a microchannel in polydimethylsiloxane (PDMS) and selected two primary bottom part substrates where the cells were seeded: tissue culture treated plastics and PDMS. Secondly, we investigated if functionalization of the PDMS substrate would enhance its suitability for protein adhesion and subsequent cell adhesion and spreading, while the plastic substrate was used without further modifications. Thirdly, all the substrates (plastic, functionalized or bare PDMS) were coated with four different adhesion proteins and murine small intestinal organoids were seeded into the microchannels. Fourthly, we conducted the experiments under two different culture medium conditions. Finally, we assessed the cell coverage percentage at multiple timepoints (1, 3, and 6 days), providing quantitative insights into the extent of cellular coverage and growth over time. Using this data, we performed a combined unianova analysis assessing the contribution of each independent variable and interactions of variables.

### Different substrates and functionalization impact the small intestinal organoid-derived epithelial cells ability to adhere and grow as monolayers

First, we wanted to assess the importance of PDMS functionalization. Cell lines, such as the commonly used Caco-2, colorectal adenocarcinoma line, usually do not require PDMS functionalization prior to coating of the surface with adhesion proteins. However, the more delicate primary intestinal epithelial cells have previously been seeded on functionalized surfaces.^13^ Thus, we wanted to understand whether functionalization was necessary and which functionalization agent would be preferable for primary small intestinal epithelial cells. To realize this, we functionalized the PDMS using either (3-aminopropyl)trimethoxysilane (APTMS) or polyethyleneimine-glutaraldehyde (PEIGA), both previously used but not compared.^13,15,20,22^ These chemical treatments were chosen to enhance the hydrophilicity of the PDMS surface and introduce reactive groups that facilitate stronger protein binding. To evaluate the impact of the chemical treatment on the hydrophilicity of the sample, all substrates, both functionalized and non-functionalized, were subjected to a hydrophobicity assay, which measured changes in surface wettability (ESI Fig. 1†). Regardless of the specific adhesion protein or mixture used, the contact angles consistently decreased to a range between 35° and 9°, rendering all surfaces hydrophilic and suitable for cellular adhesion. As controls we used either bare PDMS or tissue culture threated plastics. In addition, we provide evidence of chemical modification by comparing the binding of his-tagged GFP on treated and untreated surfaces (ESI Fig. 2†). Our results show that the GFP intensity is higher on the treated surfaces. This indicated that the protein was able to bind in a higher quantity to the treated surfaces compared to the untreated confirming the effectiveness of the treatment.

**Fig. 2:**
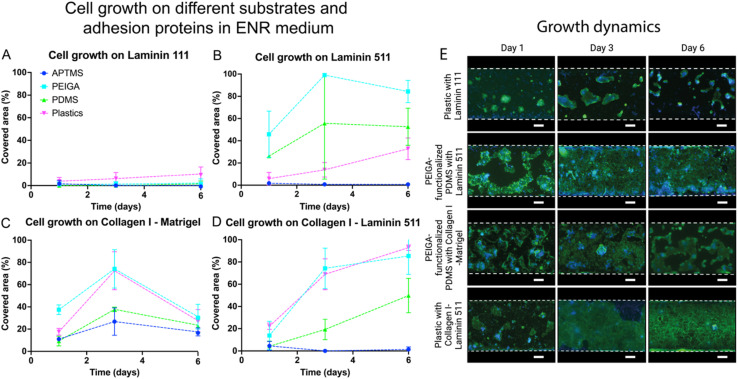
Cellular growth at different days (1, 3, 6) of organoid-derived intestinal epithelial cells on different substrates (APTMS, PEIGA, PDMS, plastic). The cells were cultured on different adhesion proteins: Laminin 111 (A), Laminin 511 (B), Collagen I and Matrigel (C), Collagen I and Laminin 511 (D) and in ENR medium. The data points represent the means and error bars the standard deviations. *N* = 3. (E) Growth dynamics showing representative images of organoid-derived intestinal epithelial cells, cultured in the microchannels as outline by the white dashed lines, of the alternative covering the largest area from (A–D) respectively (nuclei blue, actin green). Scale bar = 200 μm.

**Fig. 3:**
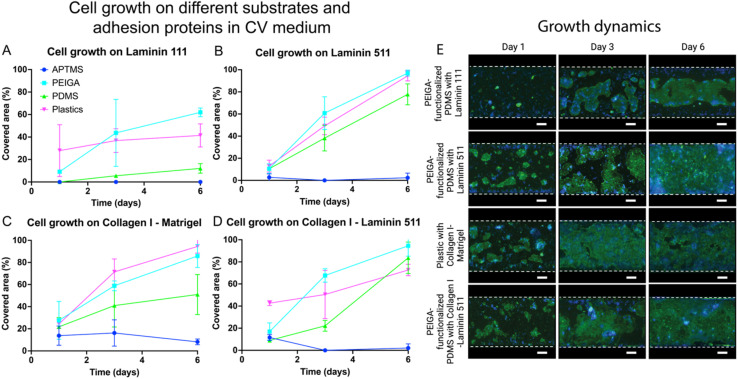
Cellular growth at different days (1, 3, 6) of organoid-derived intestinal epithelial cells on different substrates (APTMS, PEIGA, PDMS, plastic). The cells were cultured on different adhesion proteins: Laminin 111 (A), Laminin 511 (B), Collagen I and Matrigel (C), Collagen I and Laminin 511 (D) in CV medium. The data points represent the means and error bars the standard deviations. *N* = 3. (E) Growth dynamics showing representative images of organoid-derived intestinal epithelial cells, cultured in the microchannels as outline by the white dashed lines, of the alternative covering the largest area from A–D respectively (nuclei blue, actin green). Scale bar = 200 μm.

Our statistical analysis of the various substrates employed in our study revealed significant differences in their ability to support cellular adhesion and proliferation (*p* < 0.001 at day 6, ESI Tables 1, 2, 4, 5, 7, and 8†). Among the substrates tested, PEIGA-functionalized PDMS emerged as the most effective in promoting cell growth, demonstrating superior performance compared to other treatments (Fig. 2 and 3) (*p* < 0.001 compared to APTMS-functionalized PDMS and bare PDMS, and *p* = 0.002 compared to plastics). Interestingly, the results obtained with the plastic substrate were similar to those of the PEIGA-functionalized PDMS, suggesting that both surfaces are highly conducive to cellular adhesion and proliferation (ESI Tables 2, 5, 8†). In fact, both PEIGA-functionalized PDMS and plastic substrates consistently encouraged high percentages of cellular coverage, often reaching full confluence under our experimental conditions.

On the other hand, untreated PDMS, while generally following the positive trend observed with PEIGA-functionalized PDMS and plastic, showed a slower rate of cell growth and maintained a lower percentage of coverage across all conditions. This evidence highlighted that, although untreated PDMS can support intestinal epithelial cell growth, its efficiency is markedly lower compared to the treated surfaces.

The influence of APTMS-functionalized PDMS was notably less favorable for our cells. In the majority of cases, this treatment resulted in very low cell attachment rates, pointing out its inadequacy as a substrate for supporting the growth of the intestinal epithelial cells. The only scenario where APTMS-functionalized PDMS showed some promise was when a mixture of Collagen I and Matrigel was used as the adhesion protein. However, even under these conditions, the initial cell attachment could not be sustained, with a significant decline in coverage observed after three days.

Our investigation underscores the vital role of substrate functionalization in cell culture applications. PEIGA-functionalized PDMS and tissue culture treated plastic substrates demonstrate superior performance in terms of cell adhesion and coverage, emphasizing the importance of surface modifications. Conversely, APTMS-functionalized PDMS shows suboptimal impact, revealing challenges with certain functionalization methods. Furthermore, while untreated PDMS can sustain cell growth to some extent, its functionality is notably enhanced through PEIGA functionalization. These insights deepen our understanding of how substrate properties affect cell behavior, providing valuable guidance for future research in tissue engineering and related fields.

### Different adhesion proteins impact the small intestinal organoid-derived epithelial cells ability to adhere and grow as monolayers

Next, we wanted to assess the effect of different adhesion proteins and mixtures thereof on the ability of small intestine organoid-derived epithelial cells to adhere to and grow on these surfaces. Previous research on PDMS-based organs-on-a-chip devices have predominately utilized pure Matrigel or mixtures of Collagen I and Matrigel.^13,15,18^ Even though different ratios and concentrations of these have previously been studied, different adhesion proteins have not been compared. Thus, we wanted to optimize the adhesion of our cells by comparing more proteins. In addition to a mixture of Collagen I and Matrigel, we selected Laminin 111 and Laminin 511 based on prior experimental results demonstrating their efficacy in promoting cell adhesion among the tested Laminins 111, 211, 221, 411, 421, and 511 (ESI Fig. 3†). Laminin 111 is predominately expressed in the basement membrane of the intervillous regions during development.

In addition, given that Laminin 511 is expressed along the villi of the small intestine and the importance of both the laminin 5 α chain and the laminin 1 γ chain, these results were expected.^23,24^ Moreover, a combination of Collagen I and Laminin 511 was used.

Our statistical analysis comparing the different adhesion proteins and mixtures thereof, revealed significant differences in cellular growth (*p* < 0.001 at day 6, ESI Tables 1, 3, 4, 6, 7, 9,†Fig. 2 and 3). Our post hoc analysis shows that both Laminin 511 and the mixture of Collagen I and Laminin 511 are favorably supporting cell adhesion and growth compared to Laminin 111 (*p* < 0.001) and the Collagen I and Matrigel mixture (*p* < 0.001).

For small intestinal organoid-derived epithelial cells cultured on Laminin 511 and in ENR medium we observed continuous growth and spreading of the cells over time except on APTMS-functionalized surfaces, reaching a maximum peak of coverage at day 3, peaking at 99.0 ± 1.6% on PEIGA-functionalized PDMS, and declining slightly at day 6 (Fig. 2B and E). In CV medium, the cell coverage gradually increased, approaching 100% coverage at day 6 for both PEIGA-functionalized PDMS (97.0 ± 2.7%) and plastics (94.5 ± 4.5%) (Fig. 3B). The growth dynamics of the superior alternative PEIGA-functionalized PDMS is seen in Fig. 3E. Noteworthy the coverage on bare PDMS was also high (77.8 ± 9.5%)

On Collagen I and Laminin 511 and ENR medium we observed similar results as on Laminin 511 in terms of cell attachment and growth. However, in this case the spread was slower, reaching the peak confluency at day 6, with similar values, compared to Laminin 511, on both bare and PEIGA-functionalized PDMS (49.8 ± 15.4%, and 85.4 ± 16.5% respectively) respectively, but with a noteworthy increase on plastic (92.9 ± 2.5%) (Fig. 2D and E). Also, in CV medium, cells cultured on Collagen I and Laminin 511 (Fig. 3D) showed similar cell attachment and growth results compared with Laminin 511, reaching the peak of confluence at day 6, with the highest coverage on PEIGA-functionalized PDMS (94.6 ± 9.4%) and substantial coverage also on plastics (72.7 ± 5.2%) and bare PDMS (83.7 ± 14.2%). The growth dynamics of the superior alternative, PEIGA-functionalized PDMS is seen in Fig. 3E.

Notably, the results obtained with Laminin 511 and the mixture of Collagen I and Laminin 511 showed no statistically significant difference at day 6 (*p* = 0.196, ESI Table 9†), displaying very similar outcomes, indicating that both are equally supporting the small intestinal epithelial cell monolayer growth. The use of Laminin 511 as an intestinal epithelial cell adhesion protein has also been found promising in previous studies using ordinary plastic tissue culture plates.^25^

In contrast, cells cultured on Laminin 111 displayed subpar cell adhesion and growth compared to the Laminin 511-containing conditions and reached a maximum coverage of only 10.3 ± 6.2% at day 6, on the plastic substate in ENR medium (Fig. 2A and E). In CV medium the cell coverage remarkably improved reaching a maximum surface coverage on PEIGA-functionalized PDMS (62.0 ± 3.9%) at day 6. However, this was still inferior to the Laminin 511-containing conditions (Fig. 3A and E).

Collagen I combined with Matrigel did not provide better conditions either for cell attachment and growth compared to the Laminin 511-containing conditions (Fig. 2C and 3C). In ENR medium the cell coverage reached a peak at day 3, with a maximum of 74.3 ± 18.1% and 68.9 ± 13.9%, respectively, on PEIGA-functionalized PDMS and plastics, followed by a decline to 31.1 ± 11.1%, and 27.6 ± 10.0%, respectively, at day 6. The superior alternative, PEIGA-functionalized PDMS growth dynamics is seen in Fig. 2E. Finally, using Collagen I and Matrigel in combination with the CV medium, the tendency in growth and spread was continuous and constant over time, reaching a high coverage at day 3, and an increase at day 6, with PEIGA-functionalized PDMS approaching the maximum confluence (94.5 ± 6.0%) (Fig. 3E). Interestingly, the cell coverage on APTMS-functionalized PDMS in both ENR and CV media reached its highest values in combination with Collagen I and Matrigel and showed a notable initial and 3 days coverage, but not enough to maintain the proliferation and spread of the cells.

A negative control was performed by seeding intestinal cells on the devices with all types of substrates but no coating. In none of these conditions were the cells able to adhere (ESI Fig. 4†), highlighting the pivotal role of the coating for cell survival.

Our results favor the use of the more defined adhesion proteins Laminin 511, or Collagen I together with Laminin 511. Furthermore, our experiments shed light on the dynamics surrounding the Collagen I and Matrigel mixtures. Despite APTMS-functionalized PDMS showing enhanced effectiveness when combined with Collagen I and Matrigel compared to other adhesion proteins, particularly under CV culturing conditions, the ability of the Collagen I and Matrigel mixtures to support long-term small intestinal organoid-derived epithelial cell cultures appears compromised. This is evidenced by a significant decrease in cell growth after day 3 across all tested substrates. This unexpected outcome challenges the widespread acceptance of this mixture in literature and emphasizes the need for further investigation into its long-term efficacy, especially under static conditions. However, it is worth noting that when cells are cultured with CV, their growth is sustained for a longer time. This suggests that CV medium is essential for culturing intestinal stem cells on Collagen I and Matrigel. Despite these improvements the undefined nature of Matrigel may also induce substantial variations in the culture conditions that may eventually reflect in the experimental outcomes, which further supports the use of more defined culture substrates.

### Different medium compositions impact the small intestinal organoid-derived epithelial cells ability to adhere and grow as monolayers

Once we assessed the effect of the substrate, as well as the effect of the type of coating on the cellular attachment and growth over time, we studied the effect of different cell culture media, to achieve an overall optimized protocol to culture small intestinal organoid-derived epithelial cells in 2D monolayers. To do so, epithelial cells were cultured in a conventional way as 2D monolayers in presence of medium complemented with the epidermal growth factors EGF, Noggin and R-spondin1 (ENR medium). For comparison, we cultured our small intestinal epithelial cells in a medium designed to promote the proliferation of the small intestinal epithelial stem cells. In this medium, the ENR medium is supplemented with two small molecules (CV-medium), which are known to be involved in the signaling pathways of WNT and Notch: CHIR99021, which is an inhibitor of glycogen synthase kinase 3 (GSK-3) and stabilizes β-catenin activating the WNT signaling pathway^26^ and valproic acid, which is an inhibitor of histone deacetylases (HDAC), modulating various signaling pathways implicated in cell proliferation, differentiation, and development, including the Notch signaling pathway.^27,28^

Our statistical analysis revealed a statistically significant difference in cell area coverage between the two different media (*p* < 0.001 at day 6, ESI Tables 1, 4, 7,†Fig. 2 and 3). Comparing both media, our analysis showed that adding the two small molecules (CV media) boosts cellular growth. In fact, in this medium, on every substrate except APTMS-functionalized PDMS, the proliferation appears generally enhanced compared to cells cultured in the conventional ENR media. Moreover, the data show that the use of CV with either Laminin 511, or Collagen I together with Laminin 511 maintain a more continuous and homogeneous growth over time. Similarly, in terms of substrate, the use of CV medium together with PEIGA-functionalized PDMS showed the best results (Fig. 2 and 3).

The observation of the monolayer during the analysis highlighted a difference in the morphology of the organoids cultured in CV medium compared to the ones cultured in ENR. ESI Fig. 5† compares brightfield images of murine small intestinal organoids in both CV and ENR media at day 7. In presence of CV, the organoids tend to stay round and do not bud (ESI Fig. 5A†), while if cultured in ENR, the morphology is more irregular, with a consistent presence of buds forming their characteristic crypt-villus morphology (ESI Fig. 5B†). This marked difference seem to retrace what has been already reported in literature.^26^

**Fig. 4:**
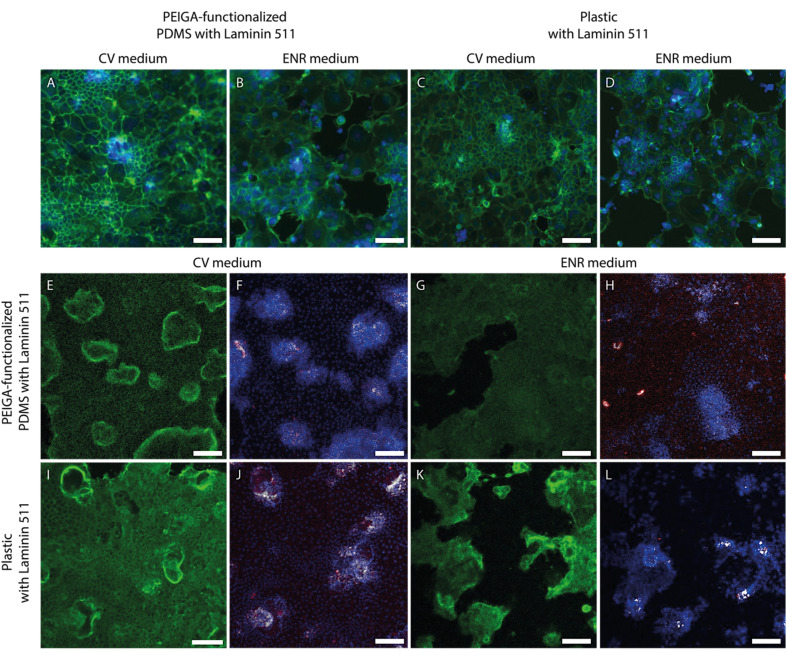
Representative images of murine small intestinal epithelium at day 6. (A) and (B) Show the epithelial coverage with actin (green) and nuclei (blue) on PEIGA-functionalized PDMS and coated with Laminin 511 in either (A) CV, or (B) ENR media. (C) and (D) Display the same epithelium on plastic coated with Laminin 511 in the presence of either (C) CV medium, or (D) ENR medium. (E–L) Display actin in green, nuclei in blue, Ki67 in red and the colocalization pixels between nuclei and Ki67 in gray. (E) and (F) Display the epithelium on PEIGA-functionalized PDMS and Laminin 511 in presence of CV, (G) and (H) display the epithelium in ENR medium. (I) and (J) Display the epithelium in plastic and Laminin 511 in presence of CV. (K) and (L) Display the epithelium in ENR medium. Scale bar = 100 μm.

To observe if these structural changes have an impact also on the 2D monolayer, the monolayer morphology was studied using actin, and Ki-67 staining, which indicates the presence and distribution of proliferative cells (Fig. 4). The morphology of the monolayer when cultured on either PEIGA-functionalized PDMS (Fig. 4A and B) or plastic (Fig. 4C and D) coated with Laminin 511 and cultured in either CV (Fig. 4A and C) or ENR (Fig. 4B and D) media display morphological differences. In the monolayers cultured in CV medium, the actin disposition seems to be more defined, with a precise spatial localization of the crypt regions, while in the monolayers culture in ENR medium the structure appears less organized, with a less evident presence of crypt regions.

The proliferating cells cultured in CV medium (Fig. 4F and J) displays an evident Ki-67 signal, located in specific points and overlapped with an accumulation of nuclei (colocalization pixels between nuclei and Ki67 in gray), suggesting the presence of crypt regions, as has previously been observed in organoid-derived monolayer cultures.^29^ In the presence of ENR medium, the Ki-67 signal is still present, although present in denser regions (Fig. 4H and L).

The discernible impact of CV medium is consistently observed as an enhancement in cellular proliferation in any condition, which may be attributed to its ability to boost cell reprogramming and proliferation, as documented in existing literature. Additionally, its capacity to improve cell survival and functionality further contributes to this effect.^26,30,31^

Furthermore, our statistical analysis revealed statically significant interactions between the substrate and the adhesion protein, the substrate and the medium, the adhesion protein and the medium, as well as the substrate, the adhesion protein and the medium (all with *p* < 0.001, ESI Tables 1, 4, and 7†). This further illustrates the importance of an optimal combination of our experimental variables for obtaining and optimal cell coverage.

### Primary human small intestinal organoid-derived epithelial cells adhere and grow under our optimized condition

Finally, to further expand the applicability of our findings, we validated the ability of human intestinal organoid-derived epithelial cells to form monolayers in our best performing identified conditions. To do so, we seeded human small intestinal organoid-derived fragments on either plastics or PEIGA-functionalized PDMS in combination with Laminin 511.

**Fig. 5:**
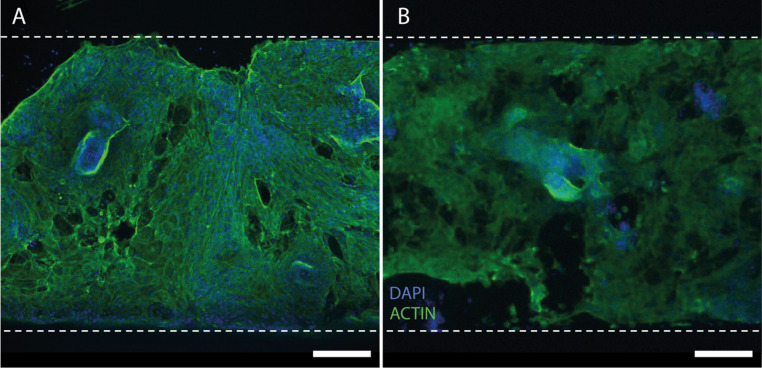
Representative images of human intestinal epithelium at day 6. (A) and (B) Show the epithelial coverage with actin (green) and nuclei (blue) in (A) plastic and (B) PDMS functionalized with PEIGA both coated with Laminin 511. The microfluidic channel is outlined by the white dashed lines. Scale bar = 200 μm.

The human epithelium was cultured for 6 days, before we assessed the cell area coverage. The epithelium cultured on plastic and PEIGA-functionalized PDMS, respectively, coated with Laminin 511 is seen in Fig. 5A and B. In both cases, the growth reached a substantial coverage, similarly to what has been observed in the murine experiments.

Here, we have provided a way to enhance the adhesion and growth of intestinal epithelial cells on PDMS and plastic surfaces commonly used in organs-on-a-chip application. Although cell adhesion and growth on these surfaces are important, other aspects such as cell heterogeneity, indicating the cell composition, or tight-junction formation, indicating the barrier tightness, should also be taken into account when choosing the most optimal culture conditions. Future work will be directed towards investigating this more thoroughly.

## Conclusions

In summary, the most optimal combination we could obtain consists of PEIGA-functionalized PDMS, Laminin 511 alone or in conjunction with Collagen I as the adhesion protein substrate, and culturing with CV medium to achieve the highest growth rate and cell coverage of small intestinal organoid-derived epithelial cells. Furthermore, the applicability of our findings in murine cells can be extended to human small intestinal organoid-derived cells. The same system allows the culture and growth of both species, facilitating the cellular adhesion and spreading over time. We anticipate that our results should be useful for future attempts in modelling the small intestine-on-a-chip using primary organoid-derived epithelial cells.

## Materials and methods

### Microfluidic device fabrication

A CNC-micro milling process was carried out to fabricate the master mold for the microfluidic device, made of PMMA (polymethacrylate). The device was designed using AutoDesk Fusion 360, and the CAD files were converted through Computer-aided manufacturing (CAM) program into numerical control (NC) programming language (G-code file). Those were used to run the CNC micro-milling machine in the Milling Machine Neo (Datron) with a 3 mm tool, at 8000 rpm spindle speed, 0.75 mm step size, 336 mm min^−1^ feed rate in XY, and 112 mm min^−1^ feed rate in Z. During the micro-milling process, some 4 mm Polymethyl methacrylate (PMMA) sheets were used as the substrates for mold fabrication. The final device consisted in a 20 mm straight channel, with 1 mm in width and 0.8 mm in height.

The devices were produced through soft lithography replication process using PDMS (Sylgard 84, Dow Corning). The PDMS was mixed in a 10 : 1 ratio for 5 minutes and degasified for 30 min in a vacuum chamber, before to be poured in the master mold and cured at 60 °C for 2 hours. After this time, the PDMS copied devices were removed from the mold and the inlet and the outlet were opened with a 1.5 mm biopsy puncher. Finally, the devices were treated with plasma at 30 W 1.50 minutes to activate the surface, and then they were either bonded to another PDMS slide or clamped to a polystyrene (plastic) coverslip (Thermo Scientific™ Nunc™ Thermanox™).

### Surface functionalization and ECM protein adhesion coating

The PDMS devices were functionalized with either APTMS((3-aminopropyl) trimethoxysilane, Sigma Aldrich Chemie GmbH #281778) or a two-steps functionalization process with PEI (polyethylenimine, Sigma Aldrich Chemie GmbH #408700) and GA (glutaraldehyde, Sigma Aldrich Chemie GmbH #G7651). In both processes, the devices were treated with plasma for 1.5 minutes. Then, for the APTMS, the devices were incubated in a 2% (v/v) dilution of APTMS in pure ethanol for 30 minutes, washed with pure ethanol three times, and finally incubated at 60 °C overnight. During the PEIGA functionalization, the devices were incubated with 1% PEI for 10 min. Then, a 0.1% solution of GA was flown for 20 min, before washing the devices three times with sterilized dH_2_O and incubated at 60 °C overnight.

After the functionalization processes, the devices were coated with ECM proteins. In the case of plastic surfaces, no previous functionalization was necessary, due to the commercial functionalization already present, that enables ECM coating without any further step. The coating was performed with two types of pure human recombinant Laminins, 511 (BioLamina) and 111 (BioLamina), and with two Collagen-based mixtures, Collagen I and Matrigel (Corning), and Collagen I and Laminin 511. Pure Laminin 511, Laminin 111 as well as the mixture of Collagen I and Laminin 511 were resuspended in 1× PBS supplemented with calcium and magnesium and the concentration of 10 μg mL^−1^ (for pure Laminins) and at the ratio of 30 μg mL^−1^ and 10 μg mL^−1^, for Collagen I-Laminin 511, respectively. Then, the devices were coated and incubated at 37 °C and 5% CO_2_ for two hours.

Collagen I and Matrigel were mixed in Dulbecco's Modified Eagle Medium (DMEM, Life Technologies #12634010) at 30 μg mL^−1^ and 1% respectively and the devices were coated and incubated for a first time for one hour at 37 °C and 5% CO_2_. After this time, the step was repeated elongating the incubation time to two hours, in the same conditions previously seen.

Laminin 111 and Laminin 511 were selected based on prior experimental results demonstrating their efficacy in promoting cell adhesion among the tested Laminins 111, 211, 221, 411, 421, and 511 (human recombinant, BioLamina). Briefly, three wells per laminin isoform of a 96 well plate were coated with 5 μg mL^−1^ of the respective laminins. To reach the desired concentration the BioLamina stocks were diluted in 1× DPBS (Ca^++^/Mg^++^). Subsequently, the well plate was sealed with Parafilm, and this was followed by an overnight incubation at 4 °C. Small intestinal organoid-derived epithelial cells were then seeded in the wells and their adhesion and coverage area was assessed at days 1 and 5.

### Hydrophobicity/hydrophilicity surface characterization

The wettability of the coated surfaces was assessed by measuring the static water contact angle (WCA) through an objective hitched to a camera (Thorlabs, DCC1645C – USB 2.0 CMOS Camera). All the coatings were repeated on the different substrates, untreated or bare, to recreate the experimental conditions, and a 10 μL dH_2_O droplet was placed on each device. Afterwards, the drop was focused with the objective, and the image was acquired and analyzed with ImageJ software, measuring with the angle tool. The angle created by the drop on the surface was measured to the left and right and the average was calculated. Angles close to 90° were considered hydrophobic, meanwhile angles between 0° and 35° were considered hydrophilic.

### Characterization of the substrate functionalization

To verify the effectiveness of PEIGA and APTMS treatments in modifying the PDMS surface, we performed a staining assay utilizing a His-tagged GFP protein. The functionalization process introduces active groups onto the PDMS surface, which can bind to the His-tagged GFP, displaying a successful modification.

We compared four different conditions: bare PDMS (untreated), bare PDMS activated with plasma, PDMS functionalized with PEIGA, and with APTMS. Each sample was incubated at room temperature for 1 hour in the dark with a 10 μg mL^−1^ solution of *Aequorea victoria* GFP His-tag Recombinant Protein (Thermo Fisher, #RP-87944). As negative control, the same experimental conditions were applied to another set of samples but without the addition of GFP.

Following incubation, all samples were thoroughly rinsed with distilled water to remove unbound GFP. The samples were then observed using epi-fluorescence inverted microscope. The fluorescence intensity, indicative of GFP binding and thus surface functionalization, was quantified using ImageJ software. The results were analyzed by comparing the fluorescence intensity across the treated and control samples, with the background intensity subtracted.

### Murine small intestinal organoid culture

Cryopreserved mouse duodenum epithelial organoids (StemCell Technologies #70931) were thawed and entrapped in Matrigel (Corning) in an appropriate volume (25 μL for one dome). The domes were placed at the center of a well in a 48-wells polystyrene plate and maintained at 37 °C and 5% CO_2_ for 30 minutes. After the polymerization of the Matrigel, each well was filled up to 250 μL with basal medium enriched with growth factor (ENR medium): Advanced D-MEM/F-12 (Life Technologies #12634010), 10 mM HEPES (Invitrogen #15630-080), 1× Glutamax (Invitrogen #35050-061), 1× penicillin, 1× streptomycin (LifeTechnolgies/Gibco, #15070-063), 50 ng mL^−1^ EGF (Peprotech #315-09), 100 ng mL^−1^ Noggin (Peprotech 250-38), and 500 ng mL^−1^ R-spondin1 (Life Technologies #100110031). The ENR medium was further supplemented with 10 μM of the ROCK inhibitor Y-27632 for the first 2 days after the seeding or after each passage. The ENR medium was changed every other day. The organoids were passaged once a week at a 1 : 3 split ratio. The domes were dissociated by mechanical stimulation in cold PBS, the old Matrigel was removed by centrifugation and washing, and the collected organoids were mechanically disrupted and resuspended in fresh Matrigel to form new domes.

### Murine small intestinal organoid-derived epithelial cell monolayer seeding and culture

In order to gather a more homogenous cellular population, all the organoids were cultured for at least one week before the seeding in the devices with CV medium, which is ENR medium complemented with the two small molecules CHIR99021 (3 μM, Stemcell technologies #100-1053) and valproic acid (1 mM Stemcell Technologies #100-1042).

The CV medium was carefully removed from each well and 300 μL of cold 1× Dulbecco's phosphate buffered saline (DPBS) supplemented with 0.1% BSA were added to each well. Three wells were combined for each device. The Matrigel domes were dissociated by pipetting, transferred in a conical tube and covered with 1× PBS-0.1% BSA before to centrifuge for 5 minutes at 4 °C at 300 g. After the removal of the supernatant, 1 mL of Recovery solution (AH diagnostics #354253) was added to the pellet. The mixture was pipetted ten times and left on ice for five minutes. After the repetition of these passages five times, the mixture was covered with 1× PBS/0.1% BSA and centrifuged again in the same conditions. Half of the pellet was resuspended in ENR and the other half in CV medium and 40 μL of cell suspension were flown into each microfluidic device. Medium was exchanged daily.

### Human organoid and monolayer culture

The research on primary human small intestinal epithelial cells was approved by the Swedish Ethical Review Authority (Dnr 2024-02048-01). Sample from patient included in this study were provided by the Biobank HUB-ICO-IDIBELL (PT20/00171), integrated in the Spanish National Biobanks Network and it was processed following standard operating procedures with the appropriate approval of the Ethics and Scientific Committees.

Cryopreserved human small intestine organoids isolated from ileum were provided by the Biobank HUB-ICO-IDIBELL. The organoids were thawed, embedded in 25 μL of Matrigel and placed in the center of a well in a 48-well polystyrene plated. After the polymerization of Matrigel at 37 °C and 5% CO_2_, the well were filled up with 250 μL of expansion medium: basal medium supplemented with 1× B-27 (Life Technologies #17504044), 10 nM Gastrin I (Merck #G9145), 1 mM Acetylcystein (Merck #A9165), 100 ng mL^−1^ Noggin (Peprotech #120-10C), 50 ng mL^−1^ EGF (Peprotech #AF-100-15), 100 ng mL^−1^ IGF-1 (Nordic Biosite #711308), 50 ng mL^−1^ FGF-2 (Peprotech #AF-100-18B), 1 μg R-spondin 1 (Peprotech #120-38), 500 nM A83 (Bio-Techne #2939/10), 0.5 nM WNT (Life Technologies #PHG0401). The expansion medium was changed every other day. The organoids were passaged once a week at a 1 : 3 split ratio; the domes were dissociated by mechanical stimulation in cold PBS, washed and resuspended in fresh Matrigel to form new domes. The organoid disaggregation and the fragment seeding in the microfluidic devices was performed according to the protocol described for the murine cells; the monolayers were cultured in expansion medium for 6 days, and the cultures were terminated at day 6.

### Staining

At each day 1, 3 and 6, the monolayers were fixed in 4% paraformaldehyde for 15 minutes, washed with 1× PBS and then permeabilized in a solution of 0.25% Triton X-100 for 20 minutes. The samples were incubated for 30 minutes with Actin Green 488 Readyprobes (Life technologies #R37110) and then washed with PBS. A counterstaining with DAPI (Merck, D9542-50MG) for 10 minutes was performed.

All the antibodies were diluted into 1% BSA. After the permeabilization, samples were washed again and blocked in 1% BSA for 40 minutes. The samples were then incubated overnight at 4 °C with anti-Ki67 antibody (Life Technologies #MA514520) at 1 : 200 dilution ratio in DPBS. DPBS was used to rinse away the unbound antibody before to incubate the Ki-67-stained samples with anti-rabbit Alexa Fluor 555 (Life Technologies # A27039). As a final step, a counterstaining with Actin Green and DAPI was performed.

### Image acquisitions and analysis of the cell area covered by the intestinal epithelial monolayers

Fluorescence microscopy images were acquired on an epi-fluorescence inverted microscope (Olympus IX73) coupled to a camera (Lumenera INFINITY2-1R) or in a confocal inverted (Nikon A1RHD). The coverage percentage was evaluated by staining the monolayer with Actin Green 488 Readyprobes (Life technologies #R37110) and acquiring pictures at different timepoints (at days 1, 3, and 6) and in three different sections of the device (on the left, on the right and in the center). The images were processed with ImageJ, calculating the covered and the empty surfaces based on the actin signal. To do so, the empty surface was identified and removed from the image. Then, the total area removed was calculated, subtracting this area from the total area of the channel, resulting in a percentage of the area covered. Once we had the covered percentage of the three different sections, the average was calculated, obtaining the total coverage in this device.

### Statistical analysis

The statistical analysis was performed using SPSS. To assess the statistical significances a unianova was employed followed by a Tukey post hoc test. The results were plotted using GraphPad Prism.

## Data availability

Data is provided by the corresponding author Maria Antfolk (maria.antfolk@bme.lth.se) upon request.

## Author contributions

F. Q., S. D., and M. A. conceptualized the work. J. T. and J. G. established and provided part of the resources. F. Q and S. D. conducted the investigations. F. Q., S. D., and M. A. performed the data analysis. M. A. acquired funding and provided supervision. F. Q., S. D., and M. A. wrote the original draft. All authors reviewed and edited the manuscript.

## Conflicts of interest

The authors declare no competing interests.

## Supplementary Material

RA-015-D4RA08290G-s001

## References

[cit1] Sato T., Vries R. G., Snippert H. J., van de Wetering M., Barker N., Stange D. E. (2009). *et al.*, Single Lgr5 stem cells build crypt-villus structures in vitro without a mesenchymal niche. Nature.

[cit2] Ootani A., Li X., Sangiorgi E., Ho Q. T., Ueno H., Toda S. (2009). *et al.*, Sustained in vitro intestinal epithelial culture within a Wnt-dependent stem cell niche. Nat. Med..

[cit3] Schweiger P. J., Jensen K. B. (2016). Modeling human disease using organotypic cultures. Curr. Opin. Cell Biol..

[cit4] Schwank G., Koo B.-K., Sasselli V., Dekkers J., Heo I., Demircan T. (2013). *et al.*, Functional repair of CFTR by CRISPR/Cas9 in intestinal stem cell organoids of cystic fibrosis patients. Cell Stem Cell.

[cit5] Ringel T., Frey N., Ringnalda F., Janjuha S., Cherkaoui S., Butz S. (2020). *et al.*, Genome-scale CRISPR screening in human intestinal organoids identifies drivers of TGF-β resistance. Cell Stem Cell.

[cit6] Michels B. E., Mosa M. H., Streibl B. I., Zhan T., Menche C., Abou-El-Ardat K. (2020). *et al.*, Pooled In Vitro and In Vivo CRISPR-Cas9 Screening Identifies Tumor Suppressors in Human Colon Organoids. Cell Stem Cell.

[cit7] Antfolk M., Jensen K. B. (2020). A bioengineering perspective on modelling the intestinal epithelial physiology in vitro. Nat. Commun..

[cit8] Co J. Y., Margalef-Català M., Li X., Mah A. T., Kuo C. J., Monack D. M. (2019). *et al.*, Controlling epithelial polarity: A human enteroid model for host-pathogen interactions. Cell Rep..

[cit9] Huh D., Matthews B. D., Mammoto A., Montoya-Zavala M., Hsin H. Y., Ingber D. E. (2010). Reconstituting organ-level lung functions on a chip. Science.

[cit10] Kim H. J., Li H., Collins J. J., Ingber D. E. (2016). Contributions of microbiome and mechanical deformation to intestinal bacterial overgrowth and inflammation in a human gut-on-a-chip. Proc. Natl. Acad. Sci. U. S. A..

[cit11] Kim H. J., Huh D., Hamilton G., Ingber D. E. (2012). Human gut-on-a-chip inhabited by microbial flora that experiences intestinal peristalsis-like motions and flow. Lab Chip.

[cit12] Kim H. J., Ingber D. E. (2013). Gut-on-a-Chip microenvironment induces human intestinal cells to undergo villus differentiation. Integr. Biol..

[cit13] Kasendra M., Tovaglieri A., Sontheimer-Phelps A., Jalili-Firoozinezhad S., Bein A., Chalkiadaki A. (2018). *et al.*, Development of a primary human Small Intestine-on-a-Chip using biopsy-derived organoids. Sci. Rep..

[cit14] Nikolaev M., Mitrofanova O., Broguiere N., Geraldo S., Dutta D., Tabata Y. (2020). *et al.*, Homeostatic mini-intestines through scaffold-guided organoid morphogenesis. Nature.

[cit15] Kasendra M., Luc R., Yin J., Manatakis D., Kulkarni G., Lucchesi C. (2020). *et al.*, Duodenum intestine-chip for preclinical drug assessment in a human relevant model. Elife.

[cit16] Gazzaniga F. S., Camacho D. M., Wu M., Silva Palazzo M. F., Dinis A. L. M., Grafton F. N. (2021). *et al.*, Harnessing Colon Chip Technology to Identify Commensal Bacteria That Promote Host Tolerance to Infection. Front. Cell. Infect. Microbiol..

[cit17] Workman M. J., Gleeson J. P., Troisi E. J., Estrada H. Q., Kerns S. J., Hinojosa C. D. (2018). *et al.*, Enhanced Utilization of Induced Pluripotent Stem Cell–Derived Human Intestinal Organoids Using Microengineered Chips. Cell. Mol. Gastroenterol. Hepatol..

[cit18] Shin W., Kim H. J. (2022). 3D in vitro morphogenesis of human intestinal epithelium in a gut-on-a-chip or a hybrid chip with a cell culture insert. Nat. Protoc..

[cit19] Shin W., Ambrosini Y. M., Shin Y. C., Wu A., Min S., Koh D. (2020). *et al.*, Robust Formation of an Epithelial Layer of Human Intestinal Organoids in a Polydimethylsiloxane-Based Gut-on-a-Chip Microdevice. Front. Biomed. Biotechnol..

[cit20] Jalili-Firoozinezhad S., Gazzaniga F. S., Calamari E. L., Camacho D. M., Fadel C. W., Bein A. (2019). *et al.*, A complex human gut microbiome cultured in an anaerobic intestine-on-a-chip. Nat. Biomed. Eng..

[cit21] Shakeri A., Khan S., Didar T. (2021). Conventional and emerging strategies for the fabrication and functionalization of PDMS-based microfluidic devices. Lab Chip.

[cit22] Laxmi S., Jianyi Y., Magdalena K., Katia K., James K., James F. (2020). *et al.*, Mechanical Stimuli Affect Escherichia coli Heat-Stable Enterotoxin-Cyclic GMP Signaling in a Human Enteroid Intestine-Chip Model. Infect. Immun..

[cit23] Fields B., DeLaForest A., Zogg M., May J., Hagen C., Komnick K. (2019). *et al.*, The Adult Murine Intestine is Dependent on Constitutive Laminin-γ1 Synthesis. Sci. Rep..

[cit24] Mahoney Z. X., Stappenbeck T. S., Miner J. H. (2008). Laminin α5 influences the architecture of the mouse small intestine mucosa. J. Cell Sci..

[cit25] Bornholdt J., Müller C. V., Nielsen M. J., Strickertsson J., Rago D., Chen Y. (2023). *et al.*, Detecting host responses to microbial stimulation using primary epithelial organoids. Gut Microbes.

[cit26] Yin X., Farin H. F., van Es J. H., Clevers H., Langer R., Karp J. M. (2014). Niche-independent high-purity cultures of Lgr5+ intestinal stem cells and their progeny. Nat. Methods.

[cit27] Greenblatt D. Y., Vaccaro A. M., Jaskula-Sztul R., Ning L., Haymart M., Kunnimalaiyaan M. (2007). *et al.*, Valproic Acid Activates Notch-1 Signaling and Regulates the Neuroendocrine Phenotype in Carcinoid Cancer Cells. Oncologist.

[cit28] Stockhausen M.-T., Sjölund J., Manetopoulos C., Axelson H. (2005). Effects of the histone deacetylase inhibitor valproic acid on Notch signalling in human neuroblastoma cells. Br. J. Cancer.

[cit29] Thorne C. A., Chen I. W., Sanman L. E., Cobb M. H., Wu L. F., Altshuler S. J. (2018). Enteroid monolayers reveal an autonomous WNT and BMP circuit controlling intestinal epithelial growth and organization. Dev. Cell.

[cit30] Aguirre-Vázquez A., Salazar-Olivo L. A., Flores-Ponce X., Arriaga-Guerrero A. L., Garza-Rodríguez D., Camacho-Moll M. E. (2021). *et al.*, 5-Aza-2′-Deoxycytidine and Valproic Acid in Combination with CHIR99021 and A83-01 Induce Pluripotency Genes Expression in Human Adult Somatic Cells. Molecules.

[cit31] Zeng J., Li Y., Ma Z., Hu M. (2021). Advances in Small Molecules in Cellular Reprogramming: Effects, Structures, and Mechanisms. Curr. Stem Cell Res. Ther..

