# Supplementary material for: Enhanced small intestinal organoid-derived epithelial cell adhesion and growth in organ-on-a-chip devices
Source: RSC Adv. 2025 Feb 5;15(5):3693–703. doi: 10.1039/d4ra08290g (PMC11795259; doi:10.1039/d4ra08290g)
Supplement: RA-015-D4RA08290G-s001 [file RA-015-D4RA08290G-s001.pdf]

Supplementary Information (SI) for RSC Advances.
This journal is © The Royal Society of Chemistry 2025

Supplementary Information

Enhanced Small Intestinal Organoid-Derived Epithelial Cell Adhesion and Growth in Organ-on-a-Chip Devices

Federica Quacquarelli*[1], Sergio Davila*[1], Jasin Taelman[2, 3], Jordi Guiu[2, 3], Maria Antfolk[1#]

[1] Department of Biomedical Engineering, Lund University, Lund, Sweden.
[2]Cell Plasticity and Regeneration Group, Regenerative Medicine Program, Institut d'Investigació Biomèdica de Bellvitge-IDIBELL, L'Hospitalet de Llobregat, Spain;
[3]Program for advancing the Clinical Translation of Regenerative Medicine of Catalonia, P-CMR[C], L'Hospitalet de Llobregat, Spain.

[#]Corresponding authors: Maria Antfolk (maria.antfolk@bme.lth.se),
*Authors contributed equally

The effect of the wettability on protein adhesion

Supplementary Figure 1 illustrates the contact angles observed on all the different surfaces used. Bare PDMS exhibited the highest contact angle of 88.7°, indicative of its hydrophobic nature. However, upon plasma activation, the contact angle of PDMS decreased significantly to 57.2°. Further functionalization of the plasma activated PDMS with APTMS resulted in a slight decrease in the contact angle to 74.2°, while PEIGA functionalization led to a more substantial reduction, bringing the contact angle down to 46.7°. The plastic substrate displayed a contact angle of 55.9°, indicative of mild hydrophobicity, and comparable to the contact angle of plasma activated PDMS.

Next, we examined the effects of different adhesion proteins on surface wettability. Regardless of the specific adhesion protein or mixture used, the contact angles consistently decreased to a range between 35° and 9°, rendering all surfaces hydrophilic and suitable for cellular adhesion. Although no significant differences were detected between the different coatings, the overall trend clearly demonstrated the impact of activation, functionalization, and coating on the wettability of PDMS and plastic surfaces. This was highlighted by the notable decrease in contact angles, affirming the importance of these treatments in enhancing the surfaces for cellular applications.

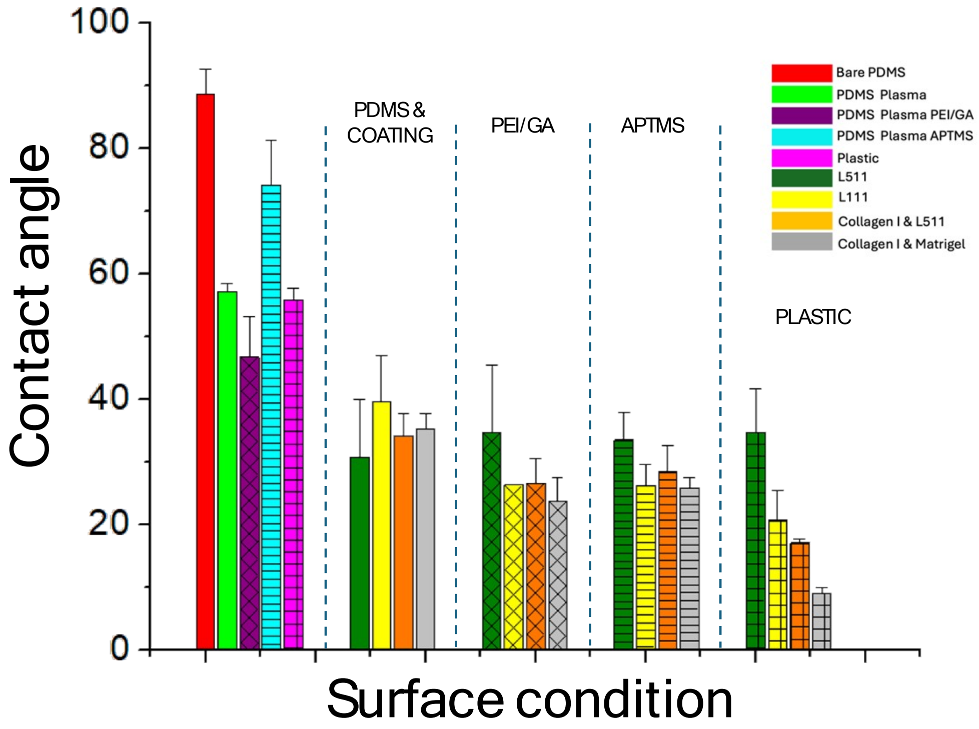

Supplementary Figure 1. Contact angle in different surfaces. The bars on the left show the contact angle on different polymers without any adhesion proteins. The bars on the right display the contact angle on functionalized and unfunctionalized substrates with adhesion proteins. Unfunctionalized PDMS with coating is shown in bars without patterns. PEIGA-functionalized PDMS is shown in bars with the crossed patterns, APTMS-functionalized PDMS is shown in bars with the straight patterns, and plastic is shown in bars with the square patterns.

Evidence of substrate functionalization

In order to provide evidence of successful substrate functionalization we utilized the fact that a His-tagged GFP protein selectively binds to the active groups available through the APTMS or PEIGA functionalization, while not binding to the hydroxyl groups present on the bare PDMS unfunctionalized surface.

We incubated both bare unfunctionalized PDMS and APTMS and PEIGA functionalized PDMS with the His-tagged GFP and subsequently measured the fluorescent intensity over the samples, while subtracting the auto-fluorescent background of the PDMS without the GFP protein. Supplementary Figure 2 shows that the fluorescent intensity of GFP is higher on both functionalized substrates indicating that successful functionalization was achieved.

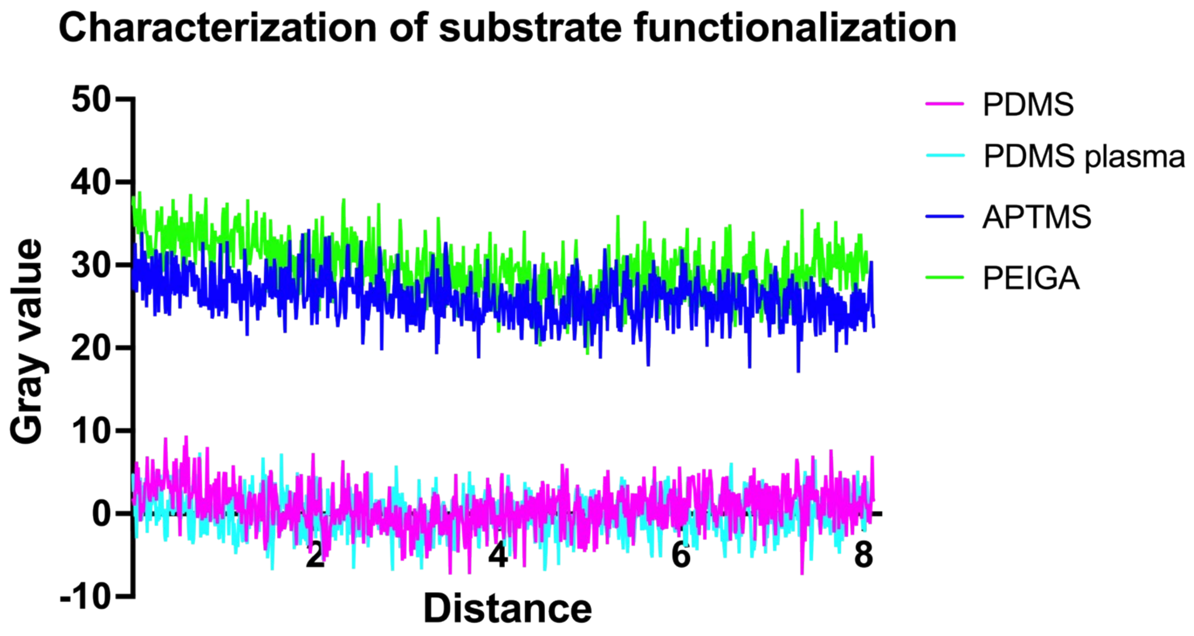

Supplementary Figure 2. Characterization of functionalization through His-tagged GFP binding. The plotted lines illustrates the fluorescent intensity of the GFP protein as measured in a line over the samples, when the auto-fluorescent background of PDMS has been subtracted.

Selection of adhesion supportive laminins

The ability to support cell adhesion and growth among a selection of laminins was assessed (Supplementary Figure 3). Human recombinant Laminins 111, 211, 221, 411, 421, and 511 were tested. On day after monolayer seeding both attachment and cell spreading can be seen on Laminins 111, 411, 421, and 511. Five days after seeding some attachment is seen on Laminins 111, 411, and 421, while on Laminin 511 there is a ≈75% confluent monolayer.

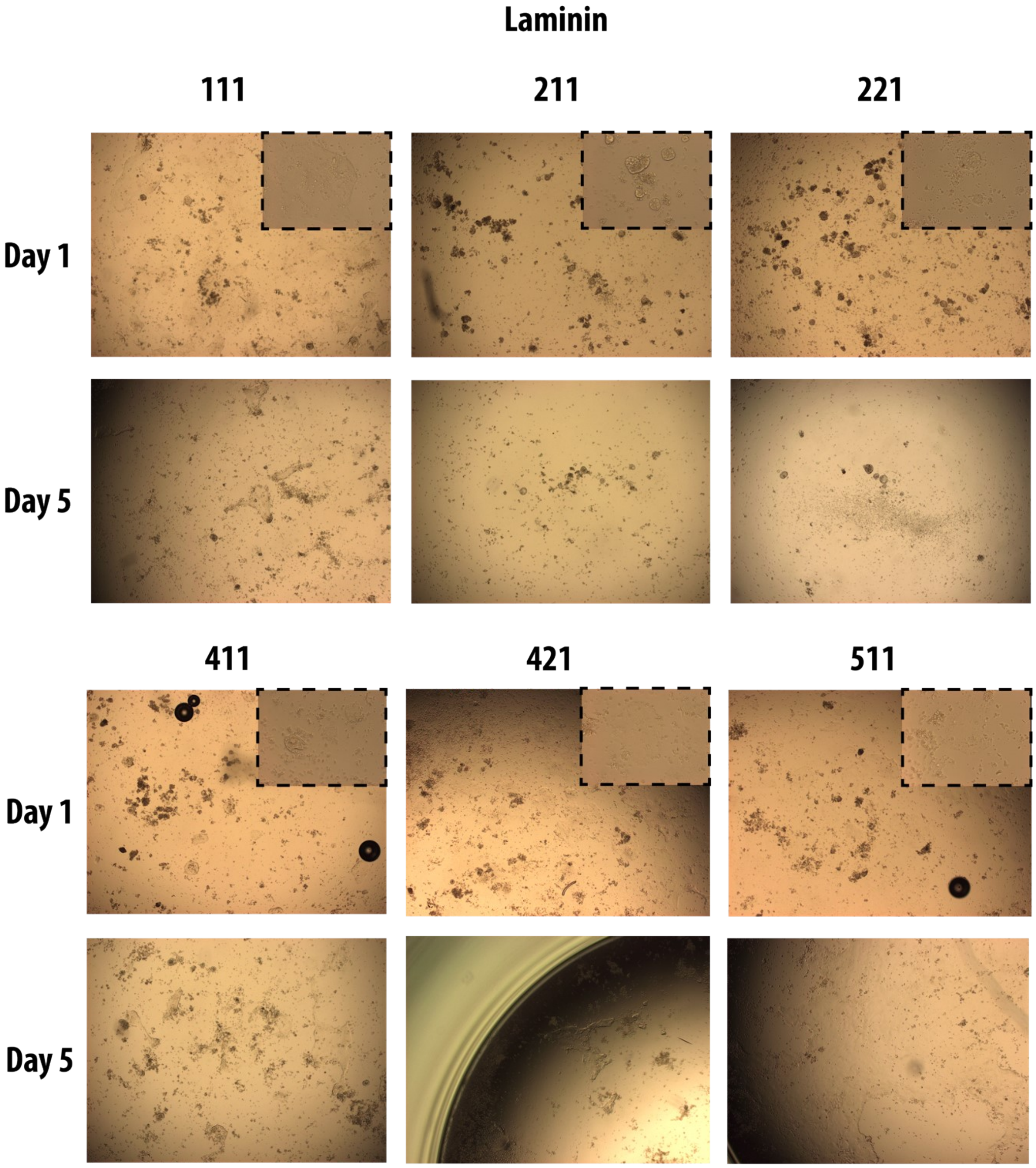

Supplementary Figure 3. Evaluation of intestinal epithelial cell adhesion to different laminins at day 1 and 5 after seeding. The image in the dashed square shows a magnification. At day 1 attachment and cell spreading is seen on laminins 111, 411, 421, and 511. At day 5 some attachment is seen on laminins 111, 411, and 421, while on laminin 511 there is a ≈75% confluent monolayer.

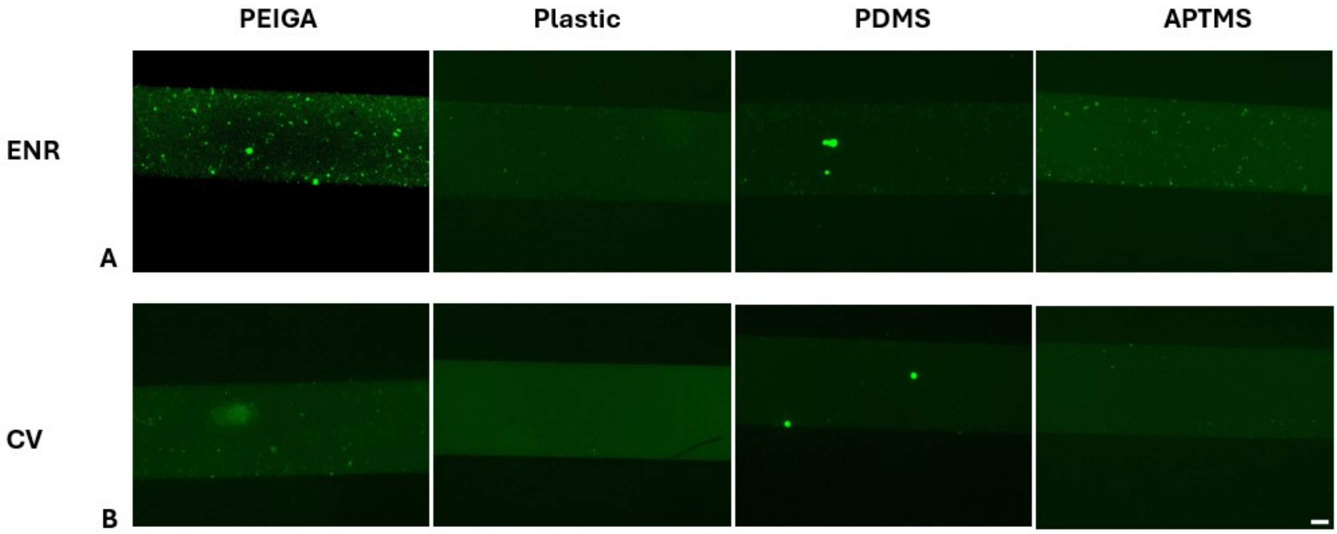

Supplementary Figure 4. Evaluation of intestinal epithelial cell growth on PDMS without coating at day 6 on PEIGA, plastic, APTMS and PDMS. A) Cells cultured in ENR medium. B) Cells cultured in CV medium (nuclei blue, actin green). In both cases, no adhesion was seen. Scale bar 200 µm.

Different medium compositions impact the small intestine organoid morphology

Supplementary Figure 5 compares brightfield images of murine small intestinal organoids in both CV and ENR media at day 7. In presence of CV, the organoids tend to stay round and do not bud (Supplementary Figure 5A), while if cultured in ENR, the morphology is more irregular, with a consistent presence of budds (Supplementary Figure 5B).

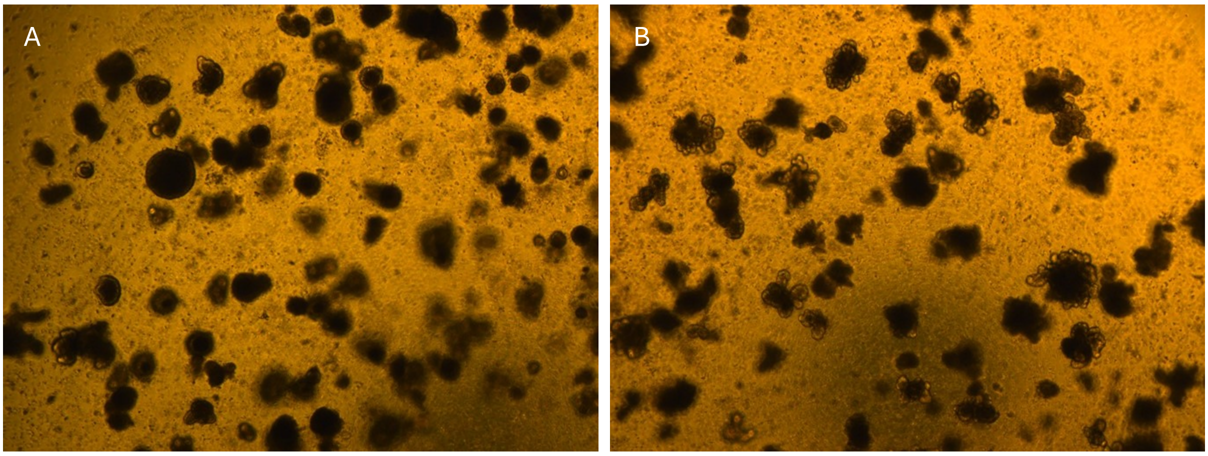

Supplementary Figure 5. Brightfield microscope images of murine intestinal organoids in different cell culture media at day 7. A) CV medium in and B) ENR medium.

Statistical analysis

Supplementary Table 1. Day 1 statistics. Results from a unianova.

Tests of Between-Subjects Effects

Dependent Variable: covered area

Source	Type III Sum of Squares	df	Mean Square	F	Sig.
Corrected Model	14445.654[a]	31	465.989	7.978	<.001
Intercept	18823.174	1	18823.174	322.260	<.001
medium	116.664	1	116.664	1.997	.162
adhesionprotein	2785.324	3	928.441	15.895	<.001
substrate	3672.725	3	1224.242	20.960	<.001
medium * adhesionprotein	1446.237	3	482.079	8.253	<.001
medium * substrate	1626.439	3	542.146	9.282	<.001
adhesionprotein * substrate	3500.113	9	388.901	6.658	<.001
medium * adhesionprotein * substrate	1298.152	9	144.239	2.469	.017
Error	3738.229	64	58.410		
Total	37007.057	96			
Corrected Total	18183.883	95			

Supplementary Table 2. Day 1 statistics. Results from a Tukey post hoc test of substrates.

Multiple Comparisons

Dependent Variable: covered area

Tukey HSD

(I) substrate	(J) substrate	Mean Difference (I-J)	Std. Error	Sig.	95% Confidence Interval Lower Bound	95% Confidence Interval Upper Bound
APTMES	PDMS	-4.21	2.21	.234	-10.03	1.61
	PEIGA	-14.44	2.21	<.001	-20.26	-8.62
	Plastic	-13.76	2.21	<.001	-19.58	-7.94
PDMS	APTMES	4.21	2.21	.234	-1.61	10.03
	PEIGA	-10.23	2.21	<.001	-16.05	-4.41
	Plastic	-9.55	2.21	<.001	-15.37	-3.73
PEIGA	APTMES	14.44	2.21	<.001	8.62	20.26
	PDMS	10.23	2.21	<.001	4.41	16.05
	Plastic	.68	2.21	.990	-5.14	6.50
Plastic	APTMES	13.76	2.21	<.001	7.94	19.58
	PDMS	9.55	2.21	<.001	3.73	15.37
	PEIGA	-.68	2.21	.990	-6.50	5.14

Supplementary Table 3. Day 1 statistics. Results from a Tukey post hoc test of adhesion proteins.

Multiple Comparisons

Dependent Variable: covered area

Tukey HSD

(I) adhesion protein	(J) adhesion protein	Mean Difference (I-J)	Std. Error	Sig.	95% Confidence Interval Lower Bound	Upper Bound
111	511	-9.09	2.21	<.001	-14.91	-3.27
	collam	-10.15	2.21	<.001	-15.97	-4.33
	colma	-14.88	2.21	<.001	-20.70	-9.06
511	111	9.09	2.21	<.001	3.27	14.91
	collam	-1.06	2.21	.963	-6.88	4.76
	colma	-5.79	2.21	.051	-11.61	.03
collam	111	10.15	2.21	<.001	4.33	15.97
	511	1.06	2.21	.963	-4.76	6.88
	colma	-4.73	2.21	.150	-10.55	1.09
colma	111	14.88	2.21	<.001	9.06	20.70
	511	5.79	2.21	.051	-.03	11.61
	collam	4.73	2.21	.150	-1.09	10.55

Supplementary Table 4. Day 3 statistics. Results from a unianova.

Tests of Between-Subjects Effects

Dependent Variable: covered area

Source	Type III Sum of Squares	df	Mean Square	F	Sig.
Corrected Model	80575.041[a]	31	2599.195	12.448	<.001
Intercept	114284.059	1	114284.059	547.332	<.001
medium	12.559	1	12.559	.060	.807
adhesionprotein	19007.808	3	6335.936	30.344	<.001
substrate	40074.888	3	13358.296	63.976	<.001
medium * adhesionprotein	2851.991	3	950.664	4.553	.006
medium * substrate	935.921	3	311.974	1.494	.225
adhesionprotein * substrate	11401.739	9	1266.860	6.067	<.001
medium * adhesionprotein * substrate	5660.342	9	628.927	3.012	.005
Error	13154.529	63	208.802		
Total	210048.506	95			
Corrected Total	93729.570	94			

Supplementary Table 5. Day 3 statistics. Results from a Tukey post hoc test of substrates.

Multiple Comparisons

Dependent Variable: covered area

Tukey HSD

(I) substrate	(J) substrate	Mean Difference (I-J)	Std. Error	Sig.	95% Confidence Interval	
					Lower Bound	Upper Bound
APTMES	PDMS	-22.01	4.17	<.001	-33.02	-10.99
	PEIGA	-54.51	4.17	<.001	-65.52	-43.50
	Plastic	-42.12	4.22	<.001	-53.25	-30.99
PDMS	APTMES	22.01	4.17	<.001	10.99	33.02
	PEIGA	-32.50	4.17	<.001	-43.51	-21.49
	Plastic	-20.11	4.22	<.001	-31.24	-8.99
PEIGA	APTMES	54.51	4.17	<.001	43.50	65.52
	PDMS	32.50	4.17	<.001	21.49	43.51
	Plastic	12.39	4.22	.023	1.26	23.52
Plastic	APTMES	42.12	4.22	<.001	30.99	53.25
	PDMS	20.11	4.22	<.001	8.99	31.24
	PEIGA	-12.39	4.22	.023	-23.52	-1.26

Supplementary Table 6. Day 3 statistics. Results from a Tukey post hoc test of adhesion proteins.

Multiple Comparisons

Dependent Variable: covered area
Tukey HSD

(I) adhesion protein	(J) adhesion protein	Mean Difference (I-J)	Std. Error	Sig.	95% Confidence Interval Lower Bound	Upper Bound
111	511	-29.15	4.22	<.001	-40.28	-18.02
	collam	-26.17	4.17	<.001	-37.18	-15.17
	colma	-38.14	4.17	<.001	-49.15	-27.13
511	111	29.15	4.22	<.001	18.02	40.28
	collam	2.98	4.22	.894	-8.15	14.10
	colma	-8.99	4.22	.154	-20.12	2.14
collam	111	26.17	4.17	<.001	15.17	37.18
	511	-2.98	4.22	.894	-14.10	8.15
	colma	-11.96	4.17	.028	-22.97	-.96
colma	111	38.14	4.17	<.001	27.13	49.15
	511	8.99	4.22	.154	-2.14	20.12
	collam	11.96	4.17	.028	.96	22.97

Supplementary Table 7. Day 6 statistics. Results from a unianova.

Tests of Between-Subjects Effects

Dependent Variable: covered area

Source	Type III Sum of Squares	df	Mean Square	F	Sig.
Corrected Model	125057.310[a]	31	4034.107	53.400	<.001
Intercept	181933.626	1	181933.626	2408.262	<.001
substrate	56842.179	3	18947.393	250.807	<.001
adhesionprotein	28049.173	3	9349.724	123.763	<.001
medium	12578.199	1	12578.199	166.498	<.001
substrate * adhesionprotein	11366.025	9	1262.892	16.717	<.001
substrate * medium	5248.207	3	1749.402	23.157	<.001
adhesionprotein * medium	2668.983	3	889.661	11.776	<.001
substrate * adhesionprotein * medium	8304.544	9	922.727	12.214	<.001
Error	4834.920	64	75.546		
Total	311825.856	96			
Corrected Total	129892.230	95			

Supplementary Table 8. Day 6 statistics. Results from a Tukey post hoc test of substrates.

Multiple Comparisons

Dependent Variable: covered area

Tukey HSD

(I) substrate	(J) substrate	Mean Difference (I-J)	Std. Error	Sig.	95% Confidence Interval Lower Bound	Upper Bound
APTMES	PDMS	-39.95	2.51	<.001	-46.57	-33.33
	PEIGA	-63.77	2.51	<.001	-70.39	-57.15
	Plastic	-54.30	2.51	<.001	-60.92	-47.68
PDMS	APTMES	39.95	2.51	<.001	33.33	46.57
	PEIGA	-23.82	2.51	<.001	-30.44	-17.20
	Plastic	-14.35	2.51	<.001	-20.97	-7.73
PEIGA	APTMES	63.77	2.51	<.001	57.15	70.39
	PDMS	23.82	2.51	<.001	17.20	30.44
	Plastic	9.47	2.51	.002	2.85	16.09
Plastic	APTMES	54.30	2.51	<.001	47.68	60.92
	PDMS	14.35	2.51	<.001	7.729	20.97
	PEIGA	-9.47	2.51	.002	-16.09	-2.85

Supplementary Table 9. Day 6 statistics. Results from a Tukey post hoc test of adhesion proteins.

Multiple Comparisons

Dependent Variable: covered area

Tukey HSD

(I) adhesion protein	(J) adhesion protein	Mean Difference (I-J)	Std. Error	Sig.	95% Confidence Interval	
					Lower Bound	Upper Bound
111	511	-39.09	2.51	<.001	-45.71	-32.47
	collam	-44.13	2.51	<.001	-50.75	-37.51
	colma	-26.22	2.51	<.001	-32.84	-19.61
511	111	39.09	2.51	<.001	32.47	45.71
	collam	-5.04	2.51	.196	-11.66	1.58
	colma	12.87	2.51	<.001	6.25	19.49
collam	111	44.13	2.51	<.001	37.51	50.75
	511	5.04	2.51	.196	-1.58	11.66
	colma	17.91	2.51	<.001	11.29	24.53
colma	111	26.22	2.51	<.001	19.61	32.84
	511	-12.87	2.51	<.001	-19.49	-6.25
	collam	-17.91	2.51	<.001	-24.53	-11.29
